# Consequences of Cannabinoid and Monoaminergic System Disruption in a Mouse Model of Autism Spectrum Disorders

**DOI:** 10.2174/157015911795017047

**Published:** 2011-03

**Authors:** E.S Onaivi, R Benno, T Halpern, M Mehanovic, N Schanz, C Sanders, X Yan, H Ishiguro, Q-R Liu, A.L Berzal, M.P Viveros, S.F Ali

**Affiliations:** 1 William Paterson University, Wayne, USA; 2 Molecular Neurobiology Branch, National Institute on Drug Abuse, NIH, Baltimore, USA; 3 Ikeda Hospital, Happoukai Medical Corporation, Ryugasaki, Japan; 4 Behavioral Neurobiology Branch, National Institute on Drug Abuse, NIH, Baltimore, USA; 5 Departamento de Fisiología (Fisiología Animal II), Facultad de Biología, Universidad Complutense, Madrid, Spain; 6 Neurochemistry Laboratory, NCTR/FDA, Jefferson, AR, USA

**Keywords:** Cannabinoid, Monoamines, Δ^9^-THC, Psychostimulants, MPTP, Behavior, Autism, BTBR T+tf/J mice.

## Abstract

Autism spectrum disorders (ASDs) are heterogenous neurodevelopmental disorders characterized by impairment in social, communication skills and stereotype behaviors. While autism may be uniquely human, there are behavioral characteristics in ASDs that can be mimicked using animal models. We used the BTBR T+tf/J mice that have been shown to exhibit autism-like behavioral phenotypes to 1). Evaluate cannabinoid-induced behavioral changes using forced swim test (FST) and spontaneous wheel running (SWR) activity and 2). Determine the behavioral and neurochemical changes after the administration of MDMA (20 mg/kg), methamphetamine (10 mg/kg) or MPTP (20 mg/kg). We found that the BTBR mice exhibited an enhanced basal spontaneous locomotor behavior in the SWR test and a reduced depressogenic profile. These responses appeared to be enhanced by the prototypic cannabinoid, Δ^9^-THC. MDMA and MPTP at the doses used did not modify SWR behavior in the BTBR mice whereas MPTP reduced SWR activity in the control CB57BL/6J mice. In the hippocampus, striatum and frontal cortex, the levels of DA and 5-HT and their metabolites were differentially altered in the BTBR and C57BL/6J mice. Our data provides a basis for further studies in evaluating the role of the cannabinoid and monoaminergic systems in the etiology of ASDs.

## INTRODUCTION

Autism is a behaviorally defined neurodevelopmental disorder characterized by impairments in social interaction and communication and repetitive/stereotyped behaviors [1, 2]. The cause of autism is not completely understood and there is no effective cure. However, genetic and environmental factors and the interaction between genes and environment are known to play a role in Autism Spectrum Disorders (ASDs) [3-7]. A common genetic variant on chromosome 5p14.1 was shown to associate with ASDs using genome-wide association studies [6] and there are currently a number of other autism susceptibility candidate genes (ASCG) that may be involved [7]. New thinking and hypothesis have been generated to include epigenetic mechanisms in ASDs [8, 9]. This is because of the complexity of ASDs and the understanding that alteration of gene function could be due to a polymorphism in DNA sequence or epigenetic programming changes of genes in the interaction with environment without change of DNA sequences [10]. 

We recognize that the symptoms of ASDs are difficult to model in rodents because of the absence of verbal communication and the variability of symptoms. Nevertheless, a number of relevant behavioral and social changes have been documented in transgenic mouse models of ASDs. Specifically mouse behavioral tests modeling some of the core symptoms of autism have now been established [11]. The goal of this study, was to use the mouse model to determine the role if any of the endocannabinoid system in autism. This was accomplished using the BTBR T+tf/J mice with autism-like behavioral phenotypes. The behavioral, morphological and neurochemical alterations in this model will allow us to test our hypothesis about the causes of autism, and may serve as an index for the evaluation of proposed treatment strategies in combination with other transgenic models. The rationale for this novel hypothesis arises from the discovery that the endocannabinoid system is one of the most abundant physiological control systems in animals and humans. This system is intricately involved with embryo development and growth with limitless interaction with most biological systems including the monoaminergic systems. The endocannabinoid system consists of genes that encode cannabinoid receptors, endogenous ligands that activate these receptors and the enzymes that synthesize, degrade and perhaps reuptake the endocannabinoids [12]. While the endocannabinoid system is ubiquitous and interacts with most biological systems, the role it plays in ASDs is unknown. We recently observed that the basal level of CB2A gene expression in the BTBR T+tf/J mice was upregulated in the cerebellum compared to control mice [13]. Therefore, we have begun studies to determine the behavioral effects of cannabinoid ligands in the BTBR mice in comparison to control groups.

## MATERIALS AND METHODS

### Animals

Adult male and female BTBR T+tf/J, C57BL/6J and 129SI/SvImJ (S129) mouse strains were housed in individual cages with access to mouse chow 12 hr in the light and 12 hr in the dark. Experiments were conducted according to standard NIH guidelines and approved by Institutional Animal Care and Use Committee.

### Drugs

Δ^9^-THC was obtained from our collaborators in NIDA intra-mural program and it was made up in a 1:1:18 solution of alcohol: emulphur: saline. MDMA, methamphetamine, and MPTP were obtained from our FDA collaborators. Animals were injected intra-peritoneal (i.p) using 1.0 and 10 mg/kg doses of Δ^9^-THC and the control animals were injected with the vehicle. The doses of MDMA (20 mg/kg), methamphetamine (10 mg/kg), MPTP (20 mg/kg) or d-amphetamine (5 mg/kg) were used. In all experiments all drugs were injected in a volume of 1ml/kg.

## EXPERIMENTAL PROCEDURE

### Motor Function Test

Spontaneous wheel running monitors were used to access motor activity and function. The standard wheel running activity monitors measures the counts per revolution and was used to access the spontaneous wheel running behavior of naïve mice and following acute treatment with the test compounds and corresponding vehicle used. The wheel running activity of the animals were monitored by the auto-counters, for 10 minutes during the assessment of spontaneous wheel running activity following specific drug pretreatment times. Data was obtained as total number of revolutions over the 10 min evaluation period. The performance of the animals following the acute administration of the test compounds to the mouse strains were compared to their respective vehicle treated controls.

### Forced Swim Test

The forced swim test (FST) paradigm was used. It consists of a glass cylinder (16 cm diameter and height 35 cm) filled to a depth 15 cm with water (23-25^o^C). One glass cylinder was used for each mouse and we tested six mice at a time using six glass cylinders and test observers. In this study a two-day swim test procedure was utilized first to access the basal performance of the different mouse strains. On the first day mice were placed in the glass cylinder with water to the specified depth, and all animals were exposed for 15-min pre-swim test prior to the 5-min forced swim test on day 2. Fresh water was introduced prior to each test. The test sessions were recorded by trained observers for consistent data recording. The observer used stop watches and counters to record immobility times and counts respectively. The data recorded during the 5-min test session were the times the animals were immobile and also the number of immobility counts during the test session. Similar data was obtained for the vehicle treated naïve control animals. During the test session the duration of immobility was defined by the animal’s stationary position, and only made the minimal movements necessary to keep the head above water.

### Neurochemical Analysis of Dopamine (DA) and Serotonin (5HT) and their Metabolites in Selected Brain Areas

Prior to preparation of animals for selected brain region dissection for neurochemical analysis, animals were scheduled for three saline or three drug injections that were given about 8 hrs apart for one day only. Mice in the different groups were injected with saline (n = 10) or these test compounds (n = 10/per group): methamphetamine (10 mg/g); MDMA (20 mg/kg) or MPTP (20 mg/kg). After the completion of drug or vehicle administration, mice were housed one per cage for two days before the animals were sacrificed two days later, and the striatum, frontal cortex and hippocampus were dissected and frozen at -80^o^C. All frozen samples were shipped to the FDA for the neurochemical analysis. Briefly, tissues from the different groups were prepared for high performance liquid chromatography (HPLC) combined with electrochemical detection to determine dopamine (DA), 3, 4-dihydroxyphenylacetic acid (DOPAC), homovanillic acid (HVA), serotonin (5HT) and 5-hydroxyindole acetic acid (5HIAA).

## CANNABINOID GENOMIC ANALYSIS IN BTBR MICE

In a previous study Liu *et al.*, 2009, [13], during the analysis of CB2-R gene expression in different brain regions of C57BL/6 mice treated with the mixed cannabinoid agonist WIN55212-2 (2mg/kg) for 7 days, we also analyzed CB2-gene expression in non-injected BTBR mice. This was accomplished by the analysis of CB2A and CB2B gene expression in brain regions, testis and the spleen. Briefly, RNA was isolated using TRIzol reagent and cDNA synthesized using SuperScript III first strand synthesis system for RT-PCR (Invitrogen, Carlsbad, CA). The expression of CB2A and CB2B genes were compared by TaqMan real-time PCR with an ABI PRISM 7900 HT Sequence Detection System, using custom designed Fam-labeled MGB probes and primers for CB2A and CB2B (Applied Biosystems, Foster City, CA). The custom-designed mouse beta-actin Fam-labeled MGB probe was used for normalization [13].

### Statistical Analysis

Prism-3 program, version 3.02 (Graphpad Software, Inc., San Diego, CA, USA) was used for statistical analyses, including *t*-tests and analysis of variance (ANOVA). Data from motor function and forced swim tests were subjected to analysis of variance for multiple comparisons followed by Turkey’s test where appropriate. For CB1 and CB2 gene expression analysis, unpaired *t*-test was used. The accepted level of significance is P <0.05.

## RESULTS

### Effects of Δ^9^-THC, Psychostimulants and Disruption of Monoaminergic System by MPTP on Motor Activity in the Mouse Model of ASD

The naïve untreated BTBR mice exhibited an enhanced basal locomotor activity as recorded in the spontaneous wheel running test. The BTBR males had slightly higher activity than the females and the motor activity of the males of the C57BL/6J were significantly lower (p<0.05, N = 10) than the activity of the BTBR males as shown in Fig. (**1A**). The effects of d-amphetamine treatment in the three mouse strains varied, with the S129 mouse showing significant locomotor activation compared to both BTBR and C57BL/6J mice as shown in Fig. (**1B**). A similar response of male and female mice in motor activity was recorded following the acute treatment of BTBR and C57BL/6J, with methamphetamine and MDMA (Fig. (**1C**)). The motor activity of C57BL/6J male mice was significantly reduced compared to those of the BTBR mice after treatment with the dopaminergic neurotoxin MPTP as shown in Fig. (**1C**). However, the motor activity of BTBR mice when compared to those of C57BL/6J and S129 mice were significantly reduced and more sensitive to the higher dose of 10 mg/kg Δ^9^-THC used in this study as shown in Fig. (**2C**). At the doses used in this study Δ^9^-THC actually enhanced motor activity in the C57BL/6J and S129 mice which were the control background mice for the BTBR animals.

### Behavioral Effects of BTBR, C57BL/6J and S129 Mice in the Forced Swim Test after Treatment with a Cannabinoid, Δ^9^-THC:

The naïve BTBR mice demonstrated reduced immobility time and increased immobility count when compared to C57BL/6J and S129 mice in the FST, as shown in Fig. (**2A**). Surprisingly, in the FST, Δ^9^-THC at the doses used did not modify the immobility time and counts of the BTBR mice when compared to the C57BL/6J and S129 mice as shown in Fig. (**2B**).

### Neurochemical Determination of DA and 5HT Levels and their Metabolites after Treatment with Methamphetamine, MDMA and MPTP

The levels of dopamine, serotonin and their metabolites were analyzed in the striatum, frontal cortex and the hippocampus after the treatment of different strains of mice with methamphetamine, MDMA and MPTP. Data on striatal DA and 5HT levels and frontal cortex 5HT levels are presented in Fig. (**3**). In this preliminary neurochemical analysis of DA, 5HT and their metabolite levels in the striatum, frontal cortex and hippocampus after the drug treatments, the levels of these monoamines and their metabolites were differentially altered in the BTBR and C57BL/6J mice used, see Fig. (**3**). The variable levels of monoamines made it difficult to define a specific association of these changes with the underlying features in the mouse model of ASDs. There are however some striking observations that can be gleaned from the effects of the doses used in drug treatments and the analyzed comparative striatal data between BTBR and C57BL/6J mice: Methamphetamine lowered BTBR DA levels relative to controls with no effect on C57BL/6J DA levels whereas MPTP had no effect on DA levels in BTBR mice relative to their controls, but lowered C57BL/6J DA levels. On the other hand MDMA had little on no significant effect on either BTBR or C57BL/6J DA levels in comparison to their respective controls.

### Cannabinoid CB2A Gene Expression is Upregulated in BTBR Mice

We have previously shown that naive BTBR mice that have been reported to have autism-like behavioral phenotypes have an upregulated higher levels of CB2A gene expression in the cerebellum without treatment with cannabinoids. This upregulation occurred usually only after sub-acute treatment with a mixed cannabinoid agonist, WIN55212-2 in the C57BL/6J mice [13]. However, no significant changes were observed in other brain regions including frontal cortex and striatum - brain areas evaluated in the current study and the hypothalamus (data not shown). The expression level of CB2B in the mouse brain is lower than CB2A and the mRNA levels could not be reliably measured by TaqMan assay (data not shown).

## DISCUSSION

While autism may be uniquely human, we have investigated the consequences of cannabinoid and monoaminergic system disruption in the BTBR T+tf/J mice that have been shown to exhibit autism-like behavioral phenotypes. We report that the BTBR mice exhibited an enhanced basal spontaneous locomotor behavior in the spontaneous wheel running (SWR) test, a measure of locomotor activity, that was reduced by the prototypic cannabinoid, Δ^9^-THC. In addition, this enhanced spontaneous wheel running behavior was sexually dimorphic as the motor activity in the naïve male BTBR mice was significantly higher than those of the naïve male C57BL/6J mice without significant alteration in the female mice. Furthermore, the doses of the psychostimulants, d-amphetamine, methamphetamine and MDMA used in this study did not modify the SWR behavior in the BTBR mice whereas MPTP reduced SWR activity in the control CB57BL/6J mice. One characteristic of ASDs is stereotype behavior characterized by high levels of repetitive self-grooming behavior that has recently been shown to be reduced in the BTBR mice by methyl-6-phenylethynl-pyridine (MPEP) – an mGluR5 antagonist [14]. It is tempting to suggest the evaluation of Δ^9^-THC or other cannabinoids with reduced psychoactivity in irritability, tantrums and self-injurious behavior associated with autistic individuals. This is because at the low doses used in this study, only the BTBR mice were sensitive to motor depressant effects of Δ^9^-THC when compared to those of C57BL/6J and S129 mice. This hypothesis is further supported by our data showing that the BTBR mice were also insensitive to the locomotor activation induced by psychostimulants and the neurotoxic effects of MPTP when compared to those of C57BL/6J and S129 mice.

An unusual behavioral phenotype characterized by exaggerated responses to stress in the BTBR mouse has been demonstrated [15]. The study showed that the BTBR mice had increased levels of the stress hormone corticosterone following tail suspension, and a heightened anxiety response in the plus-maze test, when compared to C57BL/6J mice [15]. In our current study, there were marked strain differences in immobility times and counts in the FST model of depression and BTBR mice displayed a reduced immobility time and an enhanced immobility count compared to the control C57BL/6J and S129 mice. Curiously however, Δ^9^-THC at the doses used in this study did not modify the immobility time and counts in BTBR mice when compared to the C57BL/6J and S129 mice whose immobility times and counts were differentially modified dose dependently by Δ^9^-THC.

The cause of autism is unknown, but there has been much progress and new knowledge with the environment, epigenetics and genetic factors all playing some role in the etiology of ASDs. For example multiple gene variants and genome-wide copy number variations have been reported in children with ASDs, but not in healthy controls [16]. Data from comparative genomics of autism and schizophrenia support the hypothesis that autism and schizophrenia represent diametric conditions with regard to their genomic underpinnings and phenotypic manifestations [16]. Our data indicating that the BTBR mice have an abnormal regulation of DA functioning with an upregulated CB2A gene expression in naïve BTBR mouse of ASDs [13], and our finding indicating an increased risk of schizophrenia in patients with low CB2 receptor function [17], is in agreement with the hypothesis that autism and schizophrenia represent diametric conditions [16]. Moreover, more research needs to be done to understand the nature of the neurochemical changes recorded in our preliminary study in the hippocampus, striatum and frontal cortex, where the levels of DA and 5-HT and their metabolites were differentially altered in the BTBR and C57BL/6J mice. Thus our data provides a basis for further studies in evaluating the role of the cannabinoid and monoaminergic systems in the etiology of ASDs and whether the BTBR mice can model both schizophrenia and ASDs. 

## Figures and Tables

**Fig. (1).**
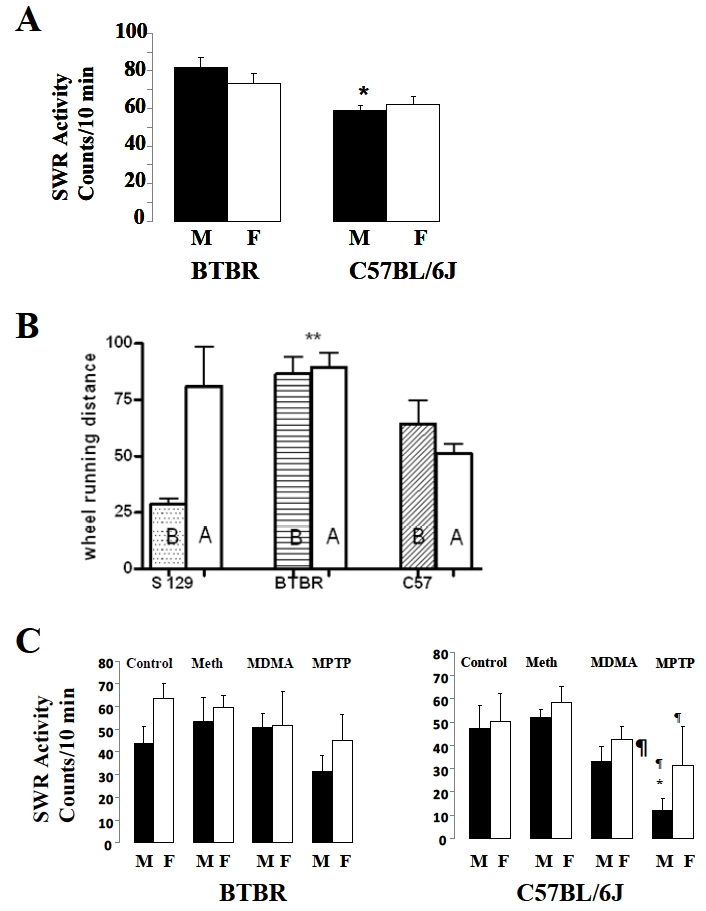
The effects of psychostimulants (d-amphetamine, Methamphetamine and MDMA), and disruption of monoaminergic system by the neurotoxin (MPTP), in a mouse model of autism spectrum disorders. Panel **A** shows the basal motor activity of male and female BTBR and C57BL/J mice in the spontaneous wheel running (SWR) monitors; panel **B** is the effect of acute 10 min treatment with d-amphetamine (5.0 mg/kg) on the performance of male BTBR and the male controls, S129 and C57BL/6J mice. Panel **C** shows the effects of acute administration of methamphetamine (10 mg/kg), MDMA (20.0 mg/kg) and MPTP (20 mg/kg) in both male and female BTBR and C57BL/6J mice in comparison to their respective controls. The duration of the wheel running behavior was accessed over a 10 min period in all animals tested. * or ¶ represents statistical significance at p<0.05 as compared to the same gender.

**Fig. (2).**
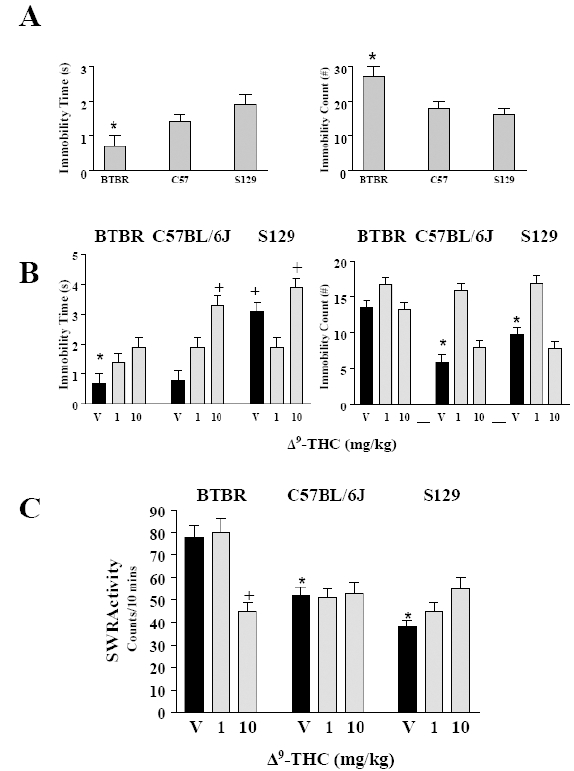
Behavioral effects of BTBR, C57BL/6J and S129 mouse strains in the FST. Panel **A** shows the basal levels of performance indicated by the time and number of immobility by the three mouse strains in the forced swim test model. Panel **B** is time and number of immobility after acute treatment of the mouse strains with Δ^9^-THC (1 and 10 mg/kg) in comparison to vehicle treated controls. Panel **C** shows the influence of acute treatment of the mouse strains with Δ^9^-THC (1 and 10 mg/kg) in the spontaneous wheel running activity monitors. * or + represents statistical significance at p<0.05 with strains and drug treatment in the behavioral measures.

**Fig. (3).**
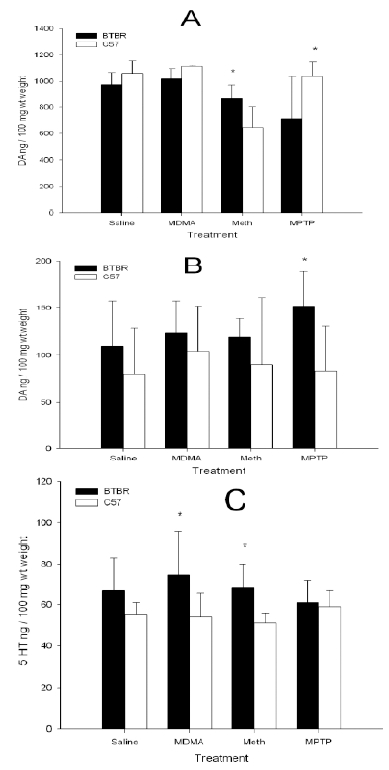
Neurochemical analysis of dopamine (DA) and serotonin (5HT) levels in striatum and frontal cortex in BTBR and C57BL/6J male and female mice following a single day three times administration of either saline, methamphetamine (10 mg/kg), MDMA (20 mg/kg) or MPTP (20 mg/kg). Regional brain areas were dissected 2 days later. Since there were no significant sex differences the data was collapsed on the variable sex. Panel **A** is the striatal dopamine level in BTBR relative to C57BL/6J mice. Panel **B** is the frontal cortex dopamine level in the two strains of mice. Panel **C** is the striatal serotonin level. *Represents statistical significance at p<0.05 using a least squares means analysis. Significance tests were performed between the two strains of mice for each of the treatments independently.
